# Early prediction of high flow nasal cannula therapy outcomes using a modified ROX index incorporating heart rate

**DOI:** 10.1186/s40560-020-00458-z

**Published:** 2020-06-22

**Authors:** Ken Junyang Goh, Hui Zhong Chai, Thun How Ong, Duu Wen Sewa, Ghee Chee Phua, Qiao Li Tan

**Affiliations:** 1 Department of Respiratory and Critical Care Medicine, Singapore General Hospital, 20 College Road, Singapore, 169856 Singapore; 2 Duke-National University of Singapore Graduate Medical School, Singapore, Singapore

**Keywords:** High flow nasal cannula, High flow oxygen therapy, Acute respiratory failure, Postextubation, Pneumonia

## Abstract

**Background:**

The ROX index (ratio of pulse oximetry/FIO_2_ to respiratory rate) has been validated to predict high flow nasal cannula therapy (HFNC) outcomes in patients with pneumonia. We evaluated a modified ROX index incorporating heart rate (HR) in patients initiated on HFNC for acute hypoxemic respiratory failure and as a preventative treatment following planned extubation.

**Methods:**

We performed a prospective observational cohort study of 145 patients treated with HFNC. ROX-HR index was defined as the ratio of ROX index over HR (beats/min), multiplied by a factor of 100. Evaluation was performed using area under the receiving operating characteristic curve (AUROC) and cutoffs assessed for prediction of HFNC failure: defined as the need for mechanical ventilation.

**Results:**

Ninety-nine (68.3%) and 46 (31.7%) patients were initiated on HFNC for acute hypoxemic respiratory failure and following a planned extubation, respectively. The majority (86.9%) of patients had pneumonia as a primary diagnosis, and 85 (56.6%) patients were immunocompromised. Sixty-one (42.1%) patients required intubation (HFNC failure). Amongst patients on HFNC for acute respiratory failure, HFNC failure was associated with a lower ROX and ROX-HR index recorded at time points between 1 and 48 h. Within the first 12 h, both indices performed with the highest AUROC at 10 h as follows: 0.723 (95% CI 0.605–0.840) and 0.739 (95% CI 0.626–0.853) for the ROX and ROX-HR index respectively. A ROX-HR index of > 6.80 was significantly associated with a lower risk of HFNC failure (hazard ratio 0.301 (95% CI 0.143–0.663)) at 10 h. This association was also observed at 2, 6, 18, and 24h, even with correction for potential confounding factors. For HFNC initiated post-extubation, only the ROX-HR index remained significantly associated with HFNC failure at all recorded time points between 1 and 24 h. A ROX-HR > 8.00 at 10 h was significantly associated with a lower risk of HFNC failure (hazard ratio 0.176 (95% CI 0.051–0.604)).

**Conclusion:**

While validation studies are required, the ROX-HR index appears to be a promising tool for early identification of treatment failure in patients initiated on HFNC for acute hypoxemic respiratory failure or as a preventative treatment after a planned extubation.

## Introduction

There is an increasing use of high flow nasal cannula oxygen therapy (HFNC) for acute hypoxemic respiratory failure, encouraged by evidence suggesting reduced intubation rates and possibly lower mortality [1, 2]. It is an attractive alternative to non-invasive ventilation (NIV) or conventional oxygen therapy because of its reported advantages in patient comfort, improved oxygenation, and decreased work of breathing in respiratory failure [3–5]. HFNC may also be used to reduce the rate of respiratory failure following a planned extubation [6–9]. Along with the growing use of HFNC, there is a need to improve early prediction of HFNC failure as delayed intubation is associated with increased mortality [10–12]. Patients identified as having a high risk of HFNC failure should be closely monitored or considered for early intubation, which may potentially improve patient outcomes.

The ROX (respiratory rate oxygenation) index, a ratio of pulse oximetry/fraction of inspired oxygen (P/F ratio) to respiratory rate per minute, has been validated to predict HFNC success in patients with pneumonia and acute respiratory failure [13, 14]. It is easily derived from commonly recorded variables measured in a non-invasive manner. However, it remains to be seen if the ROX index will perform as well in patients with respiratory failure from other causes than pneumonia, or in patients with HFNC initiated after a planned extubation. In addition, it is also unclear if the ROX index can be further improved by incorporating other vital sign parameters. Tachycardia recorded as early as 1 h into HFNC therapy has been found to be associated with HFNC failure [15]. Heart rate is a commonly measured vital sign, and incorporation into the ROX index may improve the diagnostic accuracy of the index.

In this study, we aim to evaluate the ROX index and a modified ROX index incorporating HR, in patients initiated on HFNC for hypoxemic respiratory failure and as a preventative treatment following extubation. As heart rate has an inverse relation to HFNC success, we defined the ROX-HR (respiratory rate oxygenation-heart rate) index as the ratio of ROX index over HR (beats/min) and multiplying by a factor of 100 (Figure S1).

## Methods

### Study design

We performed a prospective observational cohort study of patients initiated on HFNC (Optiflow device—MR850 heated humidified, RT202 delivery tubing and nasal cannula; Fisher & Paykel Healthcare, Auckland, New Zealand)^TM^ at a medical intensive care unit (ICU) and intermediate care area of a tertiary-care medical centre. All consecutive patients initiated on HFNC from February 2017 to September 2019 were recruited for the study. Exclusion criteria of the study were patients initiated on HFNC for bronchoscopic procedures or as a rescue therapy post-extubation, patients who were started on non-invasive ventilation after HFNC failure, and patients with a ‘do not resuscitate or intubate’ order. We obtained approval from our institutional review board for this study (CIRB Ref 2016/2988). No written consent was required in view of the purely observational nature of the study.

### HFNC protocol and management

Patients eligible for HFNC in our centre’s protocol include patients with acute hypoxemic respiratory failure, defined as having a respiratory rate > 25 breaths/min and a P/F ratio of < 300 mmHg on an oxygen device delivering 10 ≥ litres/min (LPM), in the absence of chronic respiratory failure. In our centre, HFNC may also be initiated as a preventative treatment, initiated immediately following a planned extubation. Prior to extubation, all patients had to fulfil clinical weaning criteria with a successful spontaneous breathing trial. Protocol exclusion criteria for all patients are the presence of hypercapnia (PaCO_2_ > 45 mmHg), acute respiratory failure secondary to asthma, chronic obstructive pulmonary disease (COPD) exacerbation or cardiogenic pulmonary edema, hemodynamic instability requiring vasopressor support, Glasgow coma scale (GCS) < 12 and epistaxis or recent facial or nasal surgery. HFNC was initiated at a minimum initial flow of 40 LPM. Flows were increased up to 60 LPM if required, or FIO_2_ adjusted as appropriate, with a target SpO_2_ of ≥ 92%. Discontinuation of HFNC and initiation of intubation and mechanical ventilation were based on the clinical judgement of the primary physician, guided by a protocol recommendation to consider mechanical ventilation in the presence of persistent/worsening respiratory distress, respiratory rate > 40 breaths/min, SpO_2_ < 90% for more than 5 min despite maximum flow and FIO_2_, acidemia with pH < 7.35, significant hemodynamic instability (defined as systolic blood pressure < 90 mmHg, mean arterial pressure < 65 mmHg or vasopressor requirement), deterioration in neurological status (GCS < 12) or inability to clear secretions.

### Data collection

Patient demographics, Charlson comorbidity index (CCI), clinical severity scores and arterial blood gas sampling before initiation of HFNC were recorded upon inclusion into the study. Chest radiographs (CXR) were evaluated at the beginning of HFNC therapy. The acute physiologic assessment and chronic health evaluation II (APACHE II) score and sequential organ failure assessment score (SOFA) were recorded based on the highest scores in the 24 h preceding HFNC initiation [16, 17]. The presence of chronic kidney disease (CKD) was defined as having a baseline serum creatinine of > 265 μmol/L or requiring long-term dialysis. Chronic pulmonary disease was defined as symptomatic dyspnea from a chronic respiratory condition such as COPD or interstitial lung disease. Patients were considered as immunocompromised if they had one or more of the following: haematological or solid organ malignancy, prior haematological or solid organ transplantation, human immunodeficiency (HIV) infection, liver cirrhosis with portal hypertension or receiving long-term immunosuppressive therapy. Patients were followed up until in-hospital death or discharge from hospital.

### ROX and ROX-HR index

We recorded the HR and ROX index before initiation of HFNC and at 1, 2, 4, 6, 8, 10, 12, 18, 24 and 48 h after HFNC initiation. HFNC success was defined by liberation of HFNC, and failure was defined by intubation and mechanical ventilation. Duration of HFNC was recorded as the time (h) from initiation of HFNC to successful liberation or failure. At the time of termination of HFNC, the ROX and ROX-HR index based on the latest available parameters from 1 h before termination were also recorded.

### Statistical analysis

Data are presented as number (%) for categorical variables and median (interquartile range [IQR]) for continuous variables. Data and analyses are separated based on the indication for HFNC: acute respiratory failure vs post-extubation. Patients with and without successful HFNC were compared with respect to clinical and demographic characteristics by using the Mann-Whitney *U* test for continuous variables and the chi-square test or Fisher exact test as appropriate for categorical variables. The ROX and ROX-HR index at different time points were evaluated with the area under the receiver operating characteristic curve (AUROC) for the ability to correctly classify patients as HFNC success or failure. Cutoffs for the ROX and ROX-HR index, rounded off to the nearest 0.1, were chosen to maximise the sum of sensitivity and specificity based on the receiving operating characteristic curves. From these cutoffs, Kaplan-Meier (KM) plots for HFNC failure were determined and compared using the log-rank test. Univariate and multivariate Cox proportional regression analysis was performed to evaluate the hazard ratio for cumulative probability of HFNC failure based on the ROX and ROX-HR index at different time points. Covariates that were associated with HFNC failure (*p* value of < 0.10) on univariate Cox proportional regression analysis were included in the multivariate analysis. Statistical difference was considered significant at p ≤ 0.05. All statistical analyses were performed using the SPSS software (IBM SPSS Statistics ver. 22 Chicago, IL, USA).

## Results

### Patient population and HFNC outcomes

One hundred and forty-five patients were included in the study analysis. Nineteen patients were excluded: six patients had HFNC support initiated for bronchoscopy, five patients were switched from HFNC to NIV therapy, one patient had HFNC terminated due to epistaxis, one patient had HFNC terminated for transfer to the operating theatre for surgery, and six patients had a ‘do not resuscitate or intubate’ order. Immunocompromised patients made up 56.6% (*n* = 82) of the study population. Twenty patients had recently received chemotherapy for solid organ malignancies, 28 patients had an underlying haematological malignancy or a bone marrow transplant, and 29 patients were receiving chronic immunosuppressive therapy.

Ninety-nine patients (68.3%) were initiated on HFNC for acute hypoxemic respiratory failure (Table 1). Pneumonia was the most common primary diagnosis (87/99, 87.9%). The median P/F ratio was 94 (IQR 74–138), and SOFA score was 4 (IQR 3–6) at the time of HFNC initiation. Forty-five (45.5%) patients required intubation (HFNC failure) at a median of 16 (IQR 7–36) h after HFNC initiation. HFNC failure was associated with a higher SOFA and APACHE II score (recorded as the highest score in the preceding 24 h before initiation of HFNC) and an increased proportion of CXR quadrants affected at the time of HFNC initiation (Table 1). There were no significant differences found in the proportion of immunocompromised patients or pre-HFNC arterial blood gas analysis (pH, P/F ratio, PaCO_2_ and serum bicarbonate).

**Table 1:** Comparing baseline characteristics and outcomes of patients with acute hypoxemic respiratory failure (*n* = 99)

	HFNC success (*n* = 54)	HFNC failure (*n* = 45)	*p* value
Age, years	65 (56–72)	63 (55–70)	0.171
Male gender	36 (66.7)	20 (44.4)	0.026
Charlson comorbidity index	5 (3–7)	4 (2–6)	0.086
Moderate to severe CKD	12 (22.2)	5 (11.1)	0.144
Congestive heart failure	0 (0.0)	2 (4.4)	0.204
Chronic respiratory disease	3 (10.0)	2 (7.7)	1.000
Immunocompromised host	35 (64.8)	24 (53.3)	0.246
Solid organ cancer with chemotherapy	8 (22.9)	7 (29.2)	0.585
Hematological transplant or malignancy	11 (31.4)	7 (29.2)	0.853
Chronic immunosuppressive therapy	13 (37.1)	9 (37.5)	0.978
HIV/AIDS	3 (8.6)	1 (4.2)	0.639
APACHE II*	16 (12–21)	19 (15–23)	0.011
SOFA*	4 (3–6)	5 (4–7)	0.010
Primary etiology for respiratory failure			
Pneumonia	47 (87.0)	40 (88.9)	0.779
nterstitial lung disease/drug induced pneumonitis	0 (0.0)	3 (6.7)	0.090
Cancer/Lymphangitis carcinomatosis	2 (3.7)	2 (4.4)	1.000
Others	5 (9.3)	0 (0.0)	0.061
Vasopressor support at time of HFNC initiation	1 (1.9)	0 (0.0)	1.000
Number of quadrants affected on CXR	3 (2–4)	4 (3–4)	0.013
Arterial blood gas analysis pre-HFNC initiation			
pH	7.43 (7.39–7.47)	7.44 (7.39–7.47)	0.754
PaO_2_/FIO_2_ ratio	94 (72–139)	92 (74–139)	0.697
PaCO_2,_ mmHg	36 (32–40)	34 (30–39)	0.055
Serum bicarbonate, μmol/L	24 (22–26)	23 (22–25)	0.341
Duration of HFNC, h	41.5 (22.1–70.1)	16.2 (7.4–35.5)	< 0.001
Max FIO_2_ on HFNC	80 (70–100)	100 (80–100)	0.044
Max flow on HFNC, L/min	50 (40-60)	60 (50–60)	0.084
Hospital mortality	11 (20.4)	27 (60.0)	< 0.001
ICU mortality	7 (15.2)	21 (47.7)	0.001

Values are expressed in number (percentage) and median (interquartile range). *HFNC* high flow nasal cannula, *CKD* chronic kidney disease, *HIV* human immunodeficiency, *AIDS* acquired immunodeficiency syndrome, *APACHE* acute physiologic assessment and chronic health evaluation, *SOFA* sequential organ failure assessment score, *CXR* chest x-ray, *ICU* intensive care unit. Etiology for ‘Others’ include diffuse alveolar haemorrhage, pulmonary embolism and cardiogenic pulmonary edema.

*APACHE II and SOFA scores were recorded based on the highest scores in the 24 h preceding HFNC initiation

Forty-six patients (31.7%) were initiated on HFNC post-extubation (Table 2). The median duration of mechanical ventilation prior to extubation was 114 (IQR 61–194) h. Prior to extubation, the median pH was 7.45 (IQR 7.42–7.48), with a PaCO_2_ of 41 (IQR 36–44) mmHg and P/F ratio of 164 (IQR 137–184). Sixteen (16/46, 34.8%) patients required re-intubation, at a median duration of 46 (10–87) h after HFNC initiation. Patients with HFNC failure were more likely to be immunocompromised (75% vs 37%, *p* = 0.029).

**Table 2:** Comparing baseline characteristics and outcomes of patients initiated on HFNC post extubation (*n* = 46)

	HFNC success (*n* = 30)	HFNC failure (*n* = 16)	*p* value
Age, years	64 (53–71)	61 (52–72)	0.982
Male gender	17 (56.7)	7 (43.8)	0.978
Charlson comorbidity index	5 (3–6)	4 (3–6)	0.557
Moderate to severe CKD	4 (13.3)	2 (12.5)	1.000
Congestive heart failure	1 (3.3)	2 (12.5)	0.274
Chronic respiratory disease	3 (21.4)	1 (8.3)	0.598
Immunocompromised host	11 (36.7)	12 (75.0)	0.029
Solid organ cancer with chemotherapy	4 (36.4)	1 (8.3)	0.155
Hematological transplant or malignancy	6 (54.5)	4 (33.3)	0.414
Chronic immunosuppressive therapy	1 (9.1)	6 (50.0)	0.069
HIV/AIDS	0 (0.0)	1 (8.3)	1.000
APACHE II*	15 (13–19)	16 (11–21)	0.899
SOFA*	5 (3–9)	4 (3-7)	0.368
Primary etiology for respiratory failure			
Pneumonia	25 (83.3)	14 (87.5)	1.000
Interstitial lung disease/drug induced pneumonitis	3 (10.0)	0 (0.0)	0.542
Cancer/Lymphangitis carcinomatosis	0 (0.0)	2 (12.5)	0.116
Others	2 (6.7)	0 (0.0)	0.536
Duration of mechanical ventilation before extubation, h	94 (53–197)	171 (92–194)	0.137
Vasopressor support at time of HFNC initiation	0 (0.0)	0 (0.0)	NA
Number of quadrants affected on CXR	3 (3–4)	4 (3–4)	0.327
Arterial blood gas analysis pre-HFNC initiation			
pH	7.46 (7.43–7.48)	7.45 (7.41–7.48)	0.406
PaO_2_/FIO_2_ ratio	165 153–190)	157 (129–180)	0.122
PaCO_2,_ mmHg	41 (36–44)	41 (38–45)	0.773
Serum bicarbonate, μmol/L	27 (24–30)	27 (24–31)	0.936
Duration of HFNC, h	29.3 (22.6–49.8)	46.0 (9.6–86.6)	0.827
Max FIO_2_ on HFNC	50 (50–60)	80 (60–100)	< 0.001
Max flow on HFNC, L/min	50 (40–50)	60 (50–60)	0.021
Hospital mortality	6 (20.0)	10 (62.5)	0.004
ICU mortality	4 (13.3)	8 (50.0)	0.013

Values are expressed in number (percentage) and median (interquartile range). *HFNC* high flow nasal cannula, *CKD* chronic kidney disease, *HIV* human immunodeficiency, *AIDS* acquired immunodeficiency syndrome, *APACHE* acute physiologic assessment and chronic health evaluation, *SOFA* sequential organ failure assessment score, *CXR* chest x-ray, *ICU* intensive care unit. Etiology for ‘Others’ include diffuse alveolar haemorrhage, pulmonary embolism and cardiogenic pulmonary edema.

*APACHE II and SOFA scores were recorded based on the highest scores in the 24 h preceding HFNC initiation

**Fig. 1:**
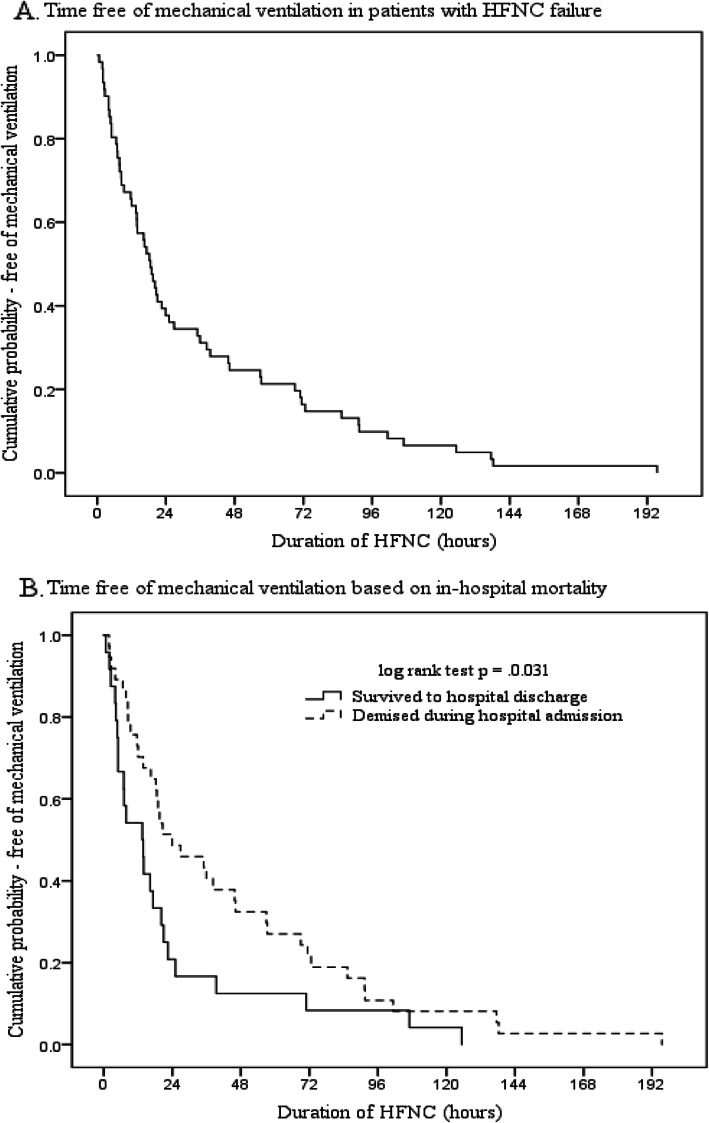
**a** Kaplan-Meier plot for the time free of mechanical ventilation in patients with HNFC failure (*n* = 61). **b** Among patients with HFNC failure, patients who did not survive had a longer duration of HFNC before intubation and mechanical ventilation

In both groups of patients (acute respiratory failure and post-extubation), HFNC failure was associated with a higher hospital and ICU mortality rate (Tables 1 and 2). Among all patients with HFNC failure, 22 (22/61, 36.1%) and 38 (38/61, 62.3%) patients were initiated on mechanical ventilation within 12 and 24 h, respectively. Initiation of mechanical ventilation after 24 h of HFNC was associated with a higher in-hospital (78.3% vs 50.0%, *p* = 0.029) and ICU mortality rate (69.6% vs 35.1%, *p* = 0.009). For the 61 patients with HFNC failure, the Kaplan-Meier plot for the probability of being free of mechanical ventilation is shown in Fig. 1a and b, which illustrates that patients who did not survive hospital admission had a longer duration of HFNC before intubation.

### Performance of the ROX and ROX-HR index

**Fig. 2:**
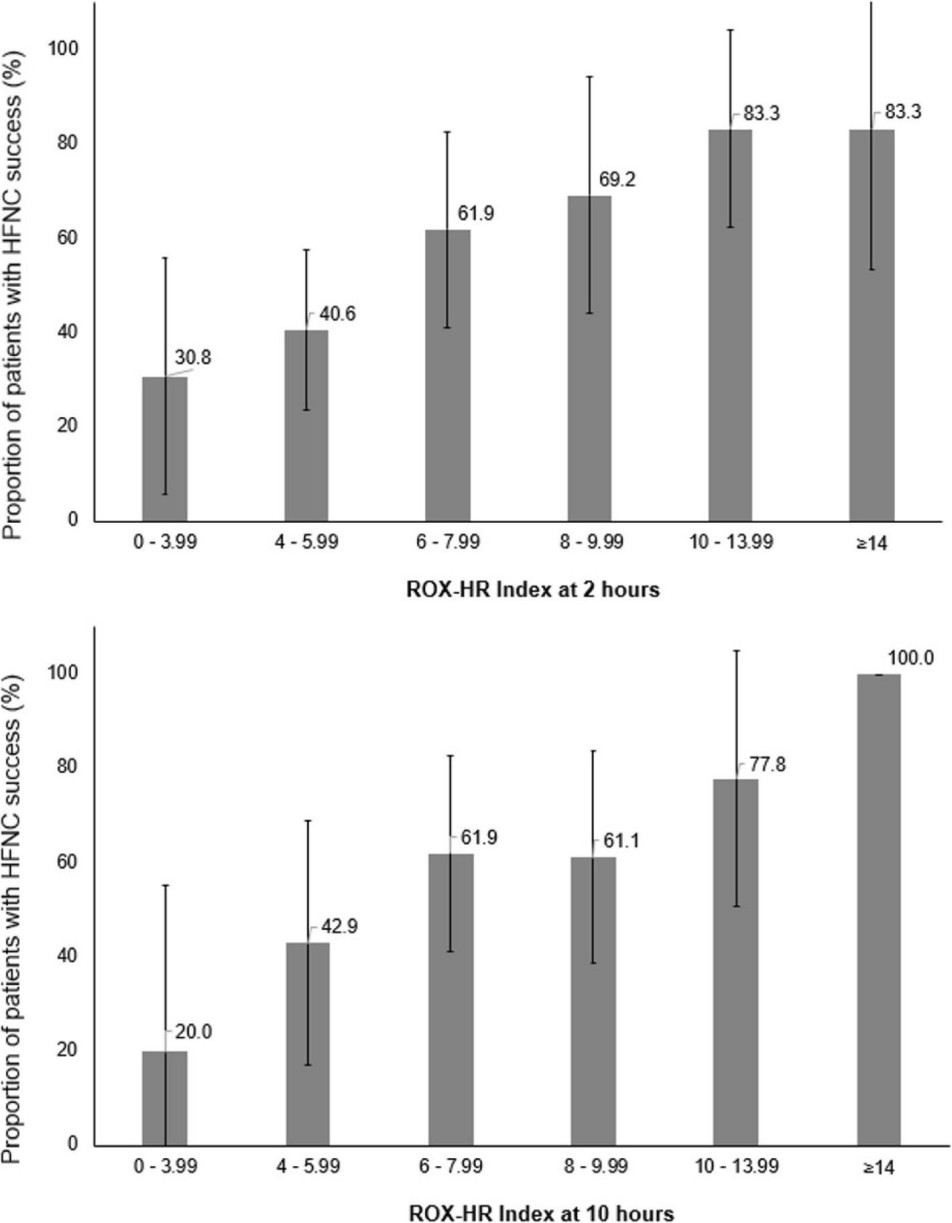
Proportion of patients with successful HFNC for acute respiratory failure, based ROX-HR index at 2 h (top graph) and 10 h (bottom graph)

In patients initiated on HFNC for acute respiratory failure, after 2, 6, 10 and 24 h, 98 (99.0%), 90 (90.9%), 83 (83.9%) and 67 (67.7%) patients remained free of mechanical ventilation, respectively (Table 3). Patients with HFNC failure had a significantly lower ROX and ROX-HR index recorded at all time points, and a significantly higher heart rate was observed at 1, 2, 4, 10 and 12 h of HFNC (Table 3). Within the first 12 h, both indices appeared to have the highest diagnostic accuracy at 10 h with an AUROC of 0.723 (95% confidence interval (CI) 0.605–0.862) and 0.739 (95% CI 0.626–0.853) for the ROX index and ROX-HR index, respectively. Figure 2 illustrates the proportion of patient with successful HFNC for acute respiratory failure, based on ROX-HR index scores at 2 and 10 h.

**Table 3:** Variables and diagnostic accuracy (for HFNC outcomes) at different time points during HFNC therapy initiated for acute respiratory failure

		Patients with HFNC success (*n* = 54)		Patients with HFNC failure (*n* = 45)		*P* value	AUROC
		Number of patients who remain on HFNC	HFNC success	Number of patients free of mechanical ventilation	HFNC failure		
ROX index	Before initiation	54	4.23 (3.50–5.13)	45	3.80 (3.12–5.37)	0.280	0.564 (0.447–0.680)
	1 h	54	5.36 (4.17–7.35)	44	4.62 (2.61–5.85)	0.032	0.625 (0.515–0.736)
	2 h	54	6.81 (5.03–8.33)	44	4.80 (3.96–6.66)	0.001	0.705 (0.602–0.809)
	4 h	53	6.26 (4.93–8.77)	40	5.23 (4.14–6.81)	0.016	0.649 (0.535–0.762)
	6 h	52	7.19 (5.47–8.58)	36	5.44 (4.42–6.66)	0.001	0.709 (0.595–0.822)
	8 h	50	7.78 (5.59–10.22)	32	6.13 (4.41–7.41)	0.013	0.667 (0.547–0.786)
	10 h	49	7.90 (5.91–9.16)	29	5.48 (4.40–7.52)	0.001	0.723 (0.605–0.840)
	12 h	49	8.25 (6.20–11.11)	27	5.66 (4.78–8.58)	0.008	0.684 (0.559–0.809)
	18 h	44	7.92 (6.56–9.98)	21	6.32 (4.65–7.51)	0.005	0.723 (0.584–0.862)
	24 h	37	8.77 (6.92–11.27)	13	5.33 (3.76–6.42)	< 0.001	0.866 (0.758–0.974)
	48 h	23	8.30 (6.53–12.83)	8	5.45 (4.50–6.55)	0.005	0.860 (0.706–1.000)
	Before termination*	NA	9.76 (8.01–12.94)	NA	4.25 (3.38–5.50)	< 0.001	NA
Heart rate (beats/min)	Before initiation	54	94 (78–110)	45	102 (88–111)	0.162	0.582 (0.486–0.696)
	1 h	54	90 (76–98)	44	100 (87–112)	0.003	0.675 (0.566–0.783)
	2 h	54	90 (77–102)	44	95 (89–112)	0.016	0.643 (0.531–0.755)
	4 h	53	89 (76–98)	40	94 (84–112)	0.011	0.658 (0.541–0.774)
	6 h	52	88 (75–96)	36	91 (78–103)	0.238	0.575 (0.449–0.702)
	8 h	50	87 (73–97)	32	89 (79–105)	0.126	0.602 (0.474–0.731)
	10 h	49	85 (72–99)	29	99 (81–109)	0.010	0.678 (0.548–0.808)
	12 h	49	83 (70–97)	27	96 (77–117)	0.041	0.642 (0.504–0.779)
	18 h	44	87 (74–100)	21	96 (71–116)	0.084	0.638 (0.469–0.806)
	24 h	37	84 (77–99)	13	103 (75–116)	0.075	0.666 (0.471–0.860)
	48 h	23	84 (72–93)	8	97 (83–111)	0.120	0.698 (0.435–0.961)
	Before termination*	NA	86 (77–95)	NA	110 (88–127)	< 0.001	NA
ROX-HR index	Before initiation	54	4.49 (3.36–6.95)	45	4.05 (2.98–5.97)	0.181	0.579 (0.464–0.693)
	1 h	54	5.97 (4.59–8.37)	44	4.76 (6.73–3.40)	0.005	0.664 (0.557–0.771)
	2 h	54	7.14 (5.58–10.75)	44	5.16 (4.02–6.97)	< 0.001	0.727 (0.627–0.828)
	4 h	53	6.85 (5.47–11.67)	40	5.83 (4.10–7.94)	0.007	0.667 (0.554–0.779)
	6 h	52	7.84 (6.51–11.36)	36	6.40 (4.55–8.50)	0.003	0.693 (0.579–0.807)
	8 h	50	8.59 (6.14–13.08)	32	7.15 (5.06–9.57)	0.011	0.670 (0.552–0.788)
	10 h	49	8.24 (7.00–12.51)	29	6.57 (4.32–8.40)	0.001	0.739 (0.626–0.853)
	12 h	49	10.44 (6.26-14.22)	27	6.38 (3.82–11.00)	0.008	0.685 (0.558–0.813)
	18 h	44	9.24 (7.74-12.17)	21	6.05 (4.38–11.40)	0.016	0.691 (0.531–0.852)
	24 h	37	10.20 (7.39-14.03)	13	6.08 (3.46–7.87)	< 0.001	0.831 (0.706–0.957)
	48 h	23	11.79 (7.07-17.53)	8	5.23 (4.81–8.69)	0.003	0.864 (0.704–1.000)
	Before termination*	NA	10.81 (8.62-15.93)	NA	4.30 (3.19–5.27)	< 0.001	NA

*HFNC* high flow nasal cannula therapy, *AUROC* area under the receiver operating characteristic curve, *NA* not applicable

*Successful or failed termination of HFNC

In patients initiated on HFNC post-extubation, 45 (97.8%), 43 (93.5%), 42 (91.3%) and 40 (87.0%) patients remained free of mechanical ventilation at 2, 6, 10 and 24 h, respectively (Table 4). Apart from the 48-h time point, patients with HFNC failure had a consistently lower ROX-HR, while no significant difference was seen with the ROX index measured at 2, 4, 8 and 18 h (Table 4). Heart rate alone predicted HFNC outcomes with an AUROC of 0.693 (95% CI 0.529–0.856) and 0.699 (95% CI 0.518–0.881) at 2 and 4 h, respectively. Within the first 12 h, the highest AUROC was found with the ROX index (0.773, 95% CI 0.617–0.928) and the ROX-HR index (0.804, 95% CI 0.660–0.948) at 10 h of HFNC therapy. Figure S2 illustrates the proportion of patient with successful HFNC after a planned extubation, based on ROX-HR index scores at 2 and 10 h.

**Table 4:** Variables and diagnostic accuracy (for HFNC outcomes) at different time points during HFNC therapy initiated after a planned extubation

		Patients with HFNC success (*n* = 30)		Patients with HFNC failure (*n* = 16)		*P* value	AUROC
		Number of patients who remain on HFNC	HFNC success	Number of patients free of mechanical ventilation	HFNC failure		
ROX index	Before initiation	30	9.22 (7.61–12.46)	16	10.30 (7.62–12.47)	0.670	0.461 (0.286–0.637)
	1 h	30	8.23 (6.58–11.44)	15	6.55 (5.61–8.91)	0.040	0.685 (0.522–0.849)
	2 h	30	8.73 (7.18–10.94)	15	7.46 (6.17–9.40)	0.177	0.626 (0.448–0.805)
	4 h	30	8.52 (6.67–962)	15	7.20 (5.81–8.18)	0.137	0.647 (0.472–0.821)
	6 h	28	8.86 (6.97–11.10)	13	6.60 (5.62–8.00)	0.015	0.754 (0.598–0.911)
	8 h	28	8.10 (7.12–11.79)	12	7.52 (5.42–8.36)	0.075	0.687 (0.514–0.860)
	10 h	28	9.06 (7.64–11.79)	12	6.43 (5.42–8.51)	0.010	0.773 (0.617–0.928)
	12 h	28	8.45 (7.70–10.27)	12	6.55 (5.42–7.42)	0.001	0.860 (0.728–0.992)
	18 h	25	9.60 (7.54–11.79)	11	7.84 (5.71–9.78)	0.137	0.665 (0.468–0.863)
	24 h	20	7.84 (6.68–10.62)	10	5.93 (5.27–7.17)	0.019	0.775 (0.561–0.990)
	48 h	12	5.82 (5.43–11.81)	7	4.86 (3.88–7.92)	0.128	0.764 (0.452–1.000)
	Before termination*	NA	12.12 (10.34–14.29)	NA	5.17 (4.21–6.40)	< 0.001	NA
Heart rate (beats/min)	Before initiation	30	90 (74–99)	16	93 (79–113)	0.299	0.594 (0.419–0.769)
	1 h	30	87 (77–95)	15	94 (77–111)	0.350	0.584 (0.397–0.771)
	2 h	30	82 (70–95)	15	95 (84–106)	0.039	0.693 (0.529–0.856)
	4 h	30	83 (72–100)	15	94 (83–115)	0.043	0.699 (0.518–0.881)
	6 h	28	81 (73–95)	13	91 (82–110)	0.182	0.640 (0.429–0.851)
	8 h	28	84 (68–96)	12	90 (86–106)	0.093	0.677 (0.497–0.856)
	10 h	28	83 (69–98)	12	90 (82–115)	0.084	0.682 (0.504–0.860)
	12 h	28	78 (70–94)	12	95 (71–119)	0.096	0.676 (0.464–0.889)
	18 h	25	80 (71–94)	11	90 (78–112)	0.142	0.663 (0.453–0.873)
	24 h	20	80 (73–100)	10	80 (74–112)	0.667	0.550 (0.320–0.781)
	48 h	12	74 (70–116)	7	90 (74–103)	0.574	0.575 (0.253–0.941)
	Before termination*	NA	80 (69–91)	NA	104 (68–119)	0.033	NA
ROX-HR index	Before initiation	30	11.54 (7.94–15.94)	16	9.96 (9.05–14.90)	0.549	0.554 (0.380–0.728)
	1 h	30	9.14 (7.72–14.56)	15	7.83 (5.90–10.98)	0.045	0.681 (0.518–0.845)
	2 h	30	10.52 (8.62–13.76)	15	8.70 (7.17–10.42)	0.041	0.690 (0.532–0.849)
	4 h	30	9.47 (7.14–12.13)	15	7.66 (5.05–10.48)	0.050	0.692 (0.512–0.873)
	6 h	28	10.05 (7.93–13.94)	13	6.87 (6.19–10.03)	0.021	0.741 (0.572–0.909)
	8 h	28	10.91 (8.03–14.70)	12	7.74 (5.42–9.30)	0.028	0.731 (0.567–0.894)
	10 h	28	10.58 (9.00–15.11)	12	7.22 (5.36–9.83)	0.004	0.804 (0.660–0.948)
	12 h	28	12.00 (8.60–13.54)	12	6.89 (5.98–7.41)	< 0.001	0.884 (0.738–1.000)
	18 h	25	11.10 (9.42–14.85)	11	8.53 (6.18–10.62)	0.034	0.735 (0.549–0.921)
	24 h	20	10.32 (7.48–13.45)	10	6.77 (5.32–8.98)	0.025	0.762 (0.547–0.977)
	48 h	12	7.89 (4.56–17.00)	7	5.63 (4.699.30)	0.423	0.639 (0.296–0.982)
	Before termination*	NA	15.79 (11.71–18.64)	NA	5.23 (4.63–7.26)	< 0.001	NA

*HFNC* High flow nasal cannula therapy, *AUROC* area under the receiver operating characteristic curve, *NA* not applicable

*Successful or failed termination of HFNC

In all patients, HFNC success was associated with a significantly higher increase in ROX-HR index from the 2 to 10 h and 6 to 10 h time points—this was not observed with the ROX index (Table S1).

### Evaluating cutoffs of the ROX-HR and ROX index for patients with acute respiratory failure

**Fig. 3:**
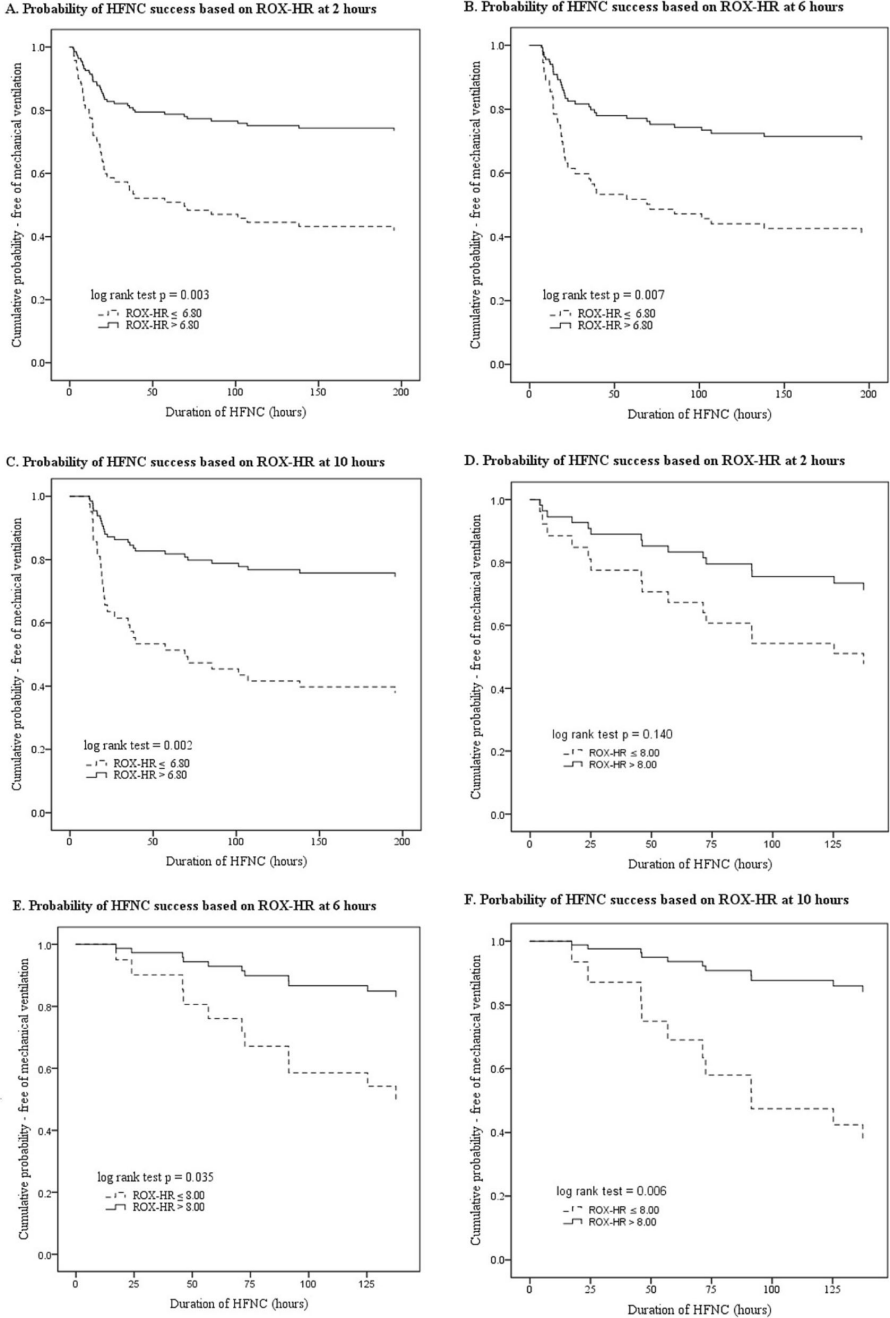
**a**–**c** Kaplan-Meier plots of HFNC success probability based on ROX-HR index at 2, 6 and 10 h for patients initiated on HFNC for acute respiratory failure. **d**, **e** Kaplan-Meier plots of HFNC success probability based on ROX-HR index at 2, 6 and 10 h for patients initiated on HFNC after a planned extubation

Using the ROC curve at 10 h into HFNC therapy, cutoffs for the ROX and ROX-HR were determined to be 5.80 and 6.80, respectively, for the prediction of HFNC success. The sensitivity, specificity, positive predictive value (PPV) and negative predictive values (NPV) of each index at 2, 6, 10, 18 and 24 h are summarised in Table 5. With Cox proportional regression analysis, a ROX-HR index of > 6.80 was associated with a lower risk of HFNC failure at all time points in the first 24 h, even after correcting for possible confounders (Gender, APACHE II score, CCI and the number of CXR quadrants involved) (Table 6). Kaplan-Meier plots illustrating significant differences in probability of HFNC success with a cutoff of 6.80 for the ROX-HR index are illustrated in Fig. 3a–c. A second cutoff of ROX < 5.00 and ROX-HR < 5.00 was determined from the ROC curves at 10 h, and their performance for the predictirised in Table S2. Compared to ROX < 5.00, a ROX-HR index < 5.00 appeared to perform with higher positive and lower negative likelihood ratios at 6, 10, 18 and 24 h.

**Table 5:** Prediction of HFNC success based on ROX and ROX-HR cut offs at different time points

	Sensitivity (%)	Specificity (%)	PPV (%)	NPV (%)	LR+	LR−
A. Prediction of HFNC success for patients initiated on HFNC for acute respiratory failure						
2-h ROX-HR > 6.80	55.56	74.41	73.17	57.14	2.17	0.60
2-h ROX > 5.80	61.11	67.44	70.21	58.00	1.87	0.58
6-h ROX-HR > 6.80	70.00	57.14	70.00	57.14	1.63	0.53
6-h ROX > 5.80	74.00	57.14	71.15	60.60	1.73	0.46
10-h ROX-HR > 6.80	78.26	58.62	75.00	62.96	1.89	0.37
10-h ROX > 5.80	78.26	58.62	75.00	62.96	1.89	0.37
18-h ROX-HR > 6.80	80.00	55.00	78.05	57.89	1.78	0.36
18-h ROX > 5.80	87.50	35.00	72.92	58.33	1.35	0.36
24-h ROX-HR > 6.80	81.82	64.29	84.39	60.00	2.29	0.28
24-h ROX > 5.80	84.85	57.14	82.35	61.54	1.98	0.27
B. Prediction of HFNC success for patients initiated on HFNC after a planned extubation						
2-h ROX-HR > 8.00	82.10	40.00	71.90	54.50	1.37	0.45
2-h ROX > 7.00	78.60	40.00	71.00	50.00	1.31	0.54
6-h ROX-HR > 8.00	74.10	63.60	83.30	50.00	2.04	0.41
6-h ROX > 7.00	74.10	54.50	80.00	46.20	1.63	0.48
10-h ROX-HR > 8.00	84.60	63.60	84.60	63.60	2.32	0.24
10-h ROX > 7.00	84.60	54.50	81.50	60.00	1.86	0.28
18-h ROX-HR > 8.00	82.60	30.00	73.10	42.90	1.18	0.58
18-h ROX > 7.00	78.30	40.00	75.00	44.40	1.31	0.54
24-h ROX-HR > 8.00	66.70	66.70	82.40	46.20	2.00	0.50
24-h ROX > 7.00	71.40	77.80	88.20	53.80	3.22	0.37

*HFNC* high flow nasal cannula therapy, *PPV* positive predictive value, *NPV* negative predictive value, *LR* likelihood ratio

**Table 6:** Cox proportional regression analysis evaluating ROX > 5.80 and ROX-HR > 6.80 for the likelihood of HFNC failure in patients initiated on HFNC for acute respiratory failure

		Univariate analysis	*p* value	Multivariate analysis	*p* value
ROX > 5.80	2 h	0.403 (0.213–0.763)	0.005	0.460 (0.238–0.892)	0.021
	6 h	0.365 (0.187–0.714)	0.003	0.494 (0.260–1.015)	0.055
	10 h	0.299 (0.142–0.626)	0.001	0.397 (0.176–0.894)	0.026
	18 h	0.300 (0.119–0.756)	0.011	0.138 (0.036–0.532)	0.004
	24 h	0.194 (0.067–0.563)	0.003	0.338 (0.101–1.136)	0.079
ROX-HR > 6.80	2 h	0.353 (0.178–0.702)	0.003	0.423 (0.211–0845)	0.015
	6 h	0.394 (0.201–0.772)	0.007	0.408 (0.201–0.828)	0.013
	10 h	0.301 (0.143–0.663)	0.002	0.369 (0.162–0.841)	0.018
	18 h	0.254 (0.105–0.616)	0.002	0.252 (0.098–0.645)	0.004
	24 h	0.177 (0.059–0.534)	0.002	0.234 (0.071–0.771)	0.017

Variables included in the multivariate analysis: gender, acute physiologic assessment and chronic health evaluation (APACHE) II, Charlson comorbidity index and number of chest x-ray quadrants involved.

We evaluated the ROX index based on previously established cutoffs of 4.88 (by Roca et al.) at 2, 6 and 12 h (Table S3 and S4) [14]. Similarly, as with a ROX index > 5.80, there was no significant association of a lower risk of HFNC failure with a ROX index ≥ 4.88 at 12 h (Table S3). Roca et al. also reported cutoffs of 2.85, 3.47 and 3.85 at 2, 6 and 12 h for the ROX index for the prediction of HFNC failure [14]. We evaluated these cutoffs in our study population, while these cutoffs performed with good specificity (> 95%), the sensitivity remained poor (< 15%). In contrast, a ROX-HR index of < 4.50 at 2 h and < 5.00 at 6 and 12 h performed with reasonable sensitivity (> 34%) and specificity (> 88%).

### Evaluating cutoffs of the ROX-HR and ROX index for patients extubated to HFNC

A cutoff of 7.00 and 8.00 was determined for the ROX and ROX-HR index, respectively, based on the ROC curves at 10 h into HFNC therapy. Table 5 summarises the sensitivity, specificity, PPV and NPV of the cutoffs at various time points within 24 h of HFNC initiation. The ROX-HR index performed with equal or higher sensitivity and specificity at 2, 6 and 10 h (Table 5). On univariate Cox proportional regression analysis, a ROX-HR index of > 8.00 was significantly associated with a lower risk of HFNC failure at 6 and 10 h, which was not seen with the ROX index >7.00 (Table 7). Kaplan-Meier plots illustrating the probability of HFNC success with a cutoff of 8.00 for the ROX-HR index at 2, 6 and 10 h are shown in Fig. 3d, e. A second cutoff of 6.00 was determined from ROC curves at 10 h, and the performance of a cutoff of ROX < 6.00 and ROX-HR < 6.00 for the prediction of HFNC failure is also summarised in Table S2.

**Table 7:** Cox proportional regression analysis evaluating ROX > 7.00 and ROX-HR > 8.00 for the prediction of HFNC failure in patients initiated on HFNC after a planned extubation

		Univariate analysis	*p* value	Multivariate analysis	*p* value
ROX > 7.00	2 h	0.519 (0.184–1.459)	0.213	0.405 (0.141–1.164)	0.093
	6 h	0.348 (0.106–1.142)	0.082	0.356 (0.108–1.171)	0.089
	10 h	0.251 (0.076–0.826)	0.023	0.287 (0.083–0.998)	0.050
	18 h	0.473 (0.133–1.680)	0.247	0.431 (0.121–1.539)	0.195
	24 h	0.169 (0.035–0.814)	0.027	0.173 (0.036–0.833)	0.029
ROX-HR > 8.00	2 h	0.459 (0.163–1.292)	0.140	0.483 (0.171–1.364)	0.169
	6 h	0.266 (0.078–0.913)	0.035	0.305 (0.086–1.079)	0.065
	10 h	0.176 (0.051–0.604)	0.006	0.194 (0.053–0.709)	0.013
	18 h	0.578 (0.149–2.238)	0.427	0.426 (0.106–1.719)	0.231
	24 h	0.296 (0.074–1.186)	0.086	0.303 (0075–1.215)	0.092

Variables included in the multivariate analysis: immunocompromised host

## Discussion

The results of this study suggest that the ROX-HR index may be a useful tool for early prediction of HFNC outcomes. This applies to patients with acute hypoxemic respiratory failure as well as patients initiated on HFNC as a preventative treatment following a planned extubation. It is easily derived from commonly recorded variables measured in a non-invasive manner and is a practical assessment tool that can be readily applied by the bedside.

For patients initiated on HFNC for acute hypoxemic respiratory failure, the ROX-HR index appears to perform consistently (AUROC > 0.65) in discriminating between HFNC success and failure at all time points. Using selected cutoffs, the ROX-HR index continues to perform well in categorising patients into low and high risk for HFNC failure, as early as 2 h into treatment. With a cutoff of ROX > 5.80 and ROX-HR > 6.80, only the ROX-HR index remained significantly associated with a lower risk of HFNC failure at all time points, after correction for possible confounders. Similarly, for patients initiated on HFNC post-extubation, the ROX-HR index remained consistently lower for patients with HFNC failure. Unlike the ROX-HR index, however, the ROX index did not appear to discriminate well between patients with HFNC success vs failure at 2, 4, 8 and 18 h.

The incorporation of the heart rate therefore appears to add value to the prediction accuracy of the ROX index. In our study, all patients with HFNC failure had a significantly higher heart rate recorded at 2 and 4 h. In patients with HFNC initiated post-extubation, HR alone recorded at 2 and 4 h achieved reasonable AUROCs (0.69 and 0.70, respectively) for the prediction of HFNC outcomes, suggesting that tachycardia, especially soon after initiation of HFNC, is associated with treatment failure. A multicentre analysis performed by Frat et al. also observed an association of HFNC failure with tachycardia, as early as 1 h into HFNC therapy [15]. An elevated heart rate may reflect an increased sympathetic drive or a decompensation of the cardiopulmonary system, and therefore be a marker for worse outcomes. Close to one third (31.7%) of our study population were patients who had HFNC initiated post-extubation. In these patients, the presence of tachycardia may also reflect an impaired cardiac reserve, which is a risk factor for the development of post-extubation respiratory failure.

The ROX-HR index also provides a means of early assessment of patients on HFNC. Early prediction of HFNC failure is crucial as most patients are intubated within 24 h of HFNC initiation (62.3% in our study) [14]. Furthermore, delayed intubation with HFNC has been shown to be associated with increased mortality [10, 14]. In our study, increased mortality was also seen in patients who were intubated after 24 h. While validation in a multicentre study is needed, the ROX-HR index appears to be a promising tool for the early identification of patients at high risk of HFNC failure.

To our knowledge, our study is also the first evaluating the use of the ROX index in patients initiated on HFNC after a planned extubation. It appears that the ROX-HR performs equally well, if not better than the ROX index in these patients. Of note, the re-intubation rate seen in our cohort (35%) is relatively higher than previous reported studies (22–23%) [7, 8]. However, there are significant differences in the study populations. Our study population was relatively more ill, with a median P/F ratio of 164, APACHE II of 15 (24 h preceding extubation) and a high proportion of immunocompromised patients (50%). This contrasts with other studies where the reported median P/F ratios (191–240) and APACHE II (median of 11) on extubation day were relatively lower. Early recognition of the need for reintubation, which is associated with worse outcomes including mortality, is an important clinical need. Indices like the ROX-HR may therefore be a useful for early assessment during the post-extubation period.

Immunocompromised patients made up more than half our study population (56.6%). There is a large interest in HFNC therapy for immunocompromised patients, with several studies suggesting that HFNC may be associated with reduced intubation rates [11, 18–20]. Previous studies have identified lower oxygenation and a higher organ dysfunction (SOFA scores) as predictive factors for HFNC failure in immunocompromised patients [12, 21]. However, there is still a paucity of evidence to guide the use of HFNC in immunocompromised patients. The substantial proportion of immunocompromised patients in our study adds strength to the applicability of the ROX-HR index to these patients.

One limitation is that this was a single centre study and conducted in a medical unit, therefore excluding surgical or post-operative patients. We also did not evaluate for the presence of atrial fibrillation or the use of beta blockers in our study. It is possible that the presence of atrial fibrillation with rapid ventricular response may by itself be a poor prognostic marker for the success of HFNC. The effects of beta-blockers on the performance of the ROX-HR index are also unclear. Furthermore, bradycardia will elevate the ROX-HR index, and if associated with hemodynamic instability, will provide physicians with a false sense of assurance—this is an important consideration when applying the ROX-HR index. In our study, 2 patients had significant bradycardia (< 50 beats/min) recorded during HFNC; both patients did not require intubation. Thirdly, evaluating a cutoff of 5.80 and 6.80, for the ROX and ROX-HR, respectively, was determined based on examination of the ROC curves of this study. Roca et al. examined the use of the ROX index for patients with acute respiratory failure from pneumonia and documented a best cutoff of 4.88 at 2, 6 and 12 h [13, 14]. Applying these cutoffs for the ROX index in our study population appeared to perform with better sensitivity compared to a ROX-HR index of > 6.80, but had poor specificity (29–51%) for HFNC success. The ROX index ≥ 4.88, when subjected to multivariate analysis, also did not appear to be consistently associated with HFNC outcomes.

Clearly, determining an ideal cutoff is challenging. Firstly, differences in study populations may lead to variation in findings. Compared to the report by Roca et al., a higher proportion of our patients on HFNC for acute respiratory failure was immunocompromised (60% vs 34%), with a higher median age (63 vs 53 years) observed in patients who failed HFNC—which may also explain the higher rate of HFNC failure seen in our study. Secondly, depending on specific clinical needs, physicians may have different priorities over the sensitivity versus specificity of the ROX or ROX-HR index, and it is likely that a “best” cutoff may also vary with differences in medical practices and ICU protocols. In our study, a lesser increase in ROX-HR index between 2 to 10 h and 6 to 10 h was also observed in patients with HFNC failure—this was not seen with the ROX index. This dynamic perspective suggests that trends in the ROX-HR index may also provide physicians with useful information. Furthermore, the absolute quantity of the index (where a ROX-HR index of < 4 or ≥ 14 is associated with a very high and low risk of HFNC failure, respectively) may also assist in clinical decision-making (Fig. 2 and S2). More studies are needed to evaluate these hypotheses carefully. Nevertheless, the ROX-HR index was consistently able to identify patients at high risk or low risk of HFNC failure based on identified cutoffs during the first 24 h into treatment. This will help to provide assurance and guidance to physicians, even in the early stages of HFNC therapy.

## Conclusion

The ROX-HR index appears to be a promising tool in the early identification of patients who are at high risk of HFNC failure and for patients initiated on HFNC for acute respiratory failure as well as a preventative strategy after a planned extubation. Larger multicentre validation studies are needed to establish the role of the ROX-HR index in patients on HFNC.

## Supplementary information

**Additional file 1: Figure S1.** Example of the ROX-HR index calculation. **Figure S2.** Proportion of patients with successful HFNC initiated after a planned extubation, based ROX-HR index at 2 hours (top graph) and 10 hours (bottom graph). **Table S1.** Comparison of the changes in ROX-HR and ROX index over different time points during HFNC. **Table S2.** Prediction of HFNC failure based on a ROX and ROX-HR cut off of < 5.00 and < 6.00 for patients initiated on HFNC for acute respiratory failure and after a planned extubation, respectively. **Table S3**. Cox proportional regression analysis evaluating ROX ≥ 4.88 for the likelihood of HFNC failure in patients with acute respiratory failure. **Table S4.** Prediction of HFNC outcomes in patients with acute respiratory failure based on ROX-HR and previously established ROX cut offs at different time points.

## Data Availability

The datasets used and/or analysed during this study are available from the corresponding author on reasonable request.

## Abbreviations

ROX: Respiratory rate oxygenation
HFNC: High flow nasal cannula therapy
HR: Heart rate
AUROC: Area under the receiver operating curve
NIV: Non-invasive ventilation
P/F: PaO_2_/FIO_2_
ICU: Intensive care unit
LPM: litres per minute
COPD: Chronic obstructive pulmonary disease
GCS: Glasgow coma scale
CCI: Charlson comorbidity index
APACHE: Acute physiology and chronic health evaluation
SOFA: Sequential organ failure assessment
CKD: Chronic kidney disease
HIV: Human immunodeficiency virus
IQR: Inter quartile range
KM: Kaplan-Meier
CI: Confidence interval
PPV: Positive predictive value
NPV: Negative predictive value
