# Supplementary material for: Early prediction of high flow nasal cannula therapy outcomes using a modified ROX index incorporating heart rate
Source: J Intensive Care. 2020 Jun 22;8:41. doi: 10.1186/s40560-020-00458-z (PMC7310118; doi:10.1186/s40560-020-00458-z)
Supplement: Supplementary file 1 — Additional file 1: Figure S1. Example of the ROX-HR index calculation. Figure S2. Proportion of patients with successful HFNC initiated after a planned extubation, based ROX-HR index at 2 hours (top graph) and 10 hours (bottom graph). Table S1. Comparison of the changes in ROX-HR and ROX index over different time points during HFNC. Table S2. Prediction of HFNC failure based on a ROX and ROX-HR cut off of < 5.00 and < 6.00 for patients initiated on HFNC for acute respiratory failure and after a planned extubation, respectively. Table S3. Cox proportional regression analysis evaluating ROX ≥ 4.88 for the likelihood of HFNC failure in patients with acute respiratory failure. Table S4. Prediction of HFNC outcomes in patients with acute respiratory failure based on ROX-HR and previously established ROX cut offs at different time points. [file 40560_2020_458_MOESM1_ESM.docx]

**Supplementary data**


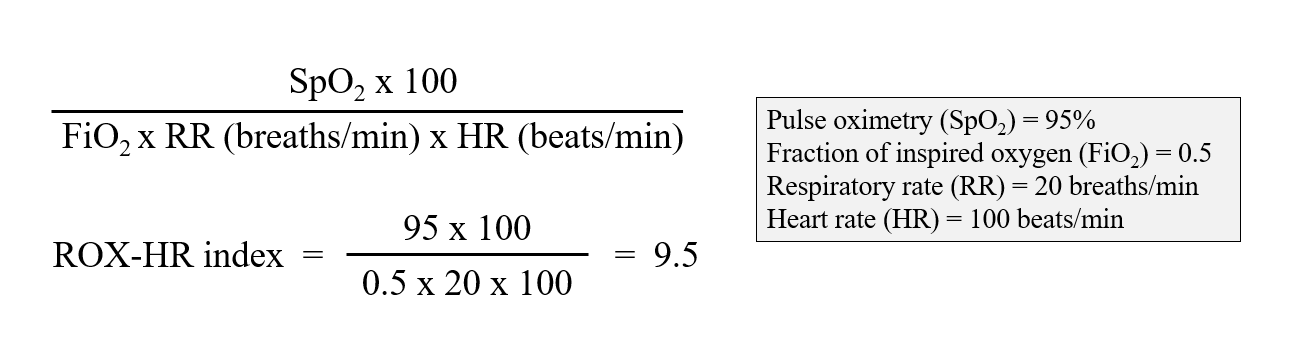


**Figure S1**. Example of the ROX-HR index calculation


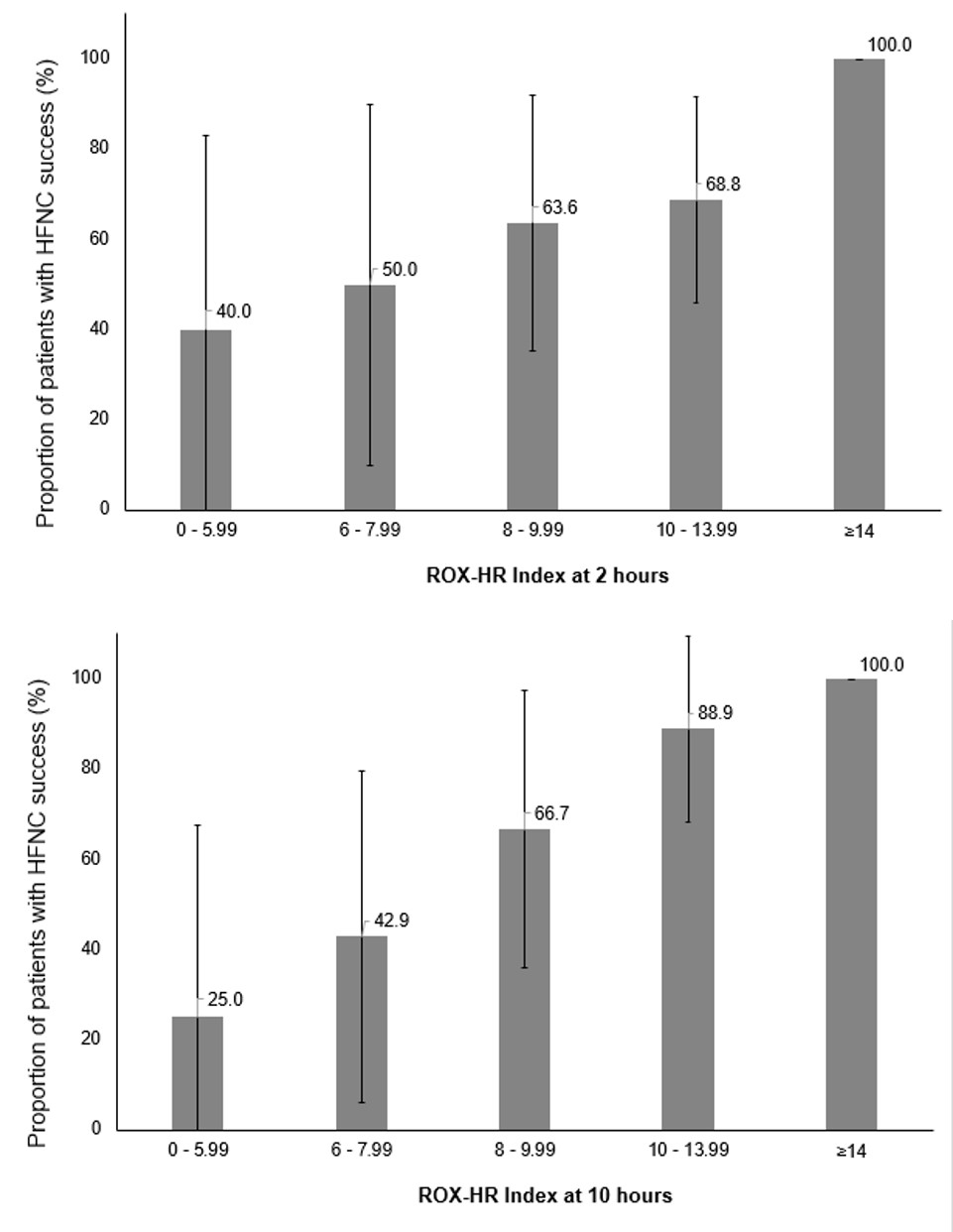


**Figure S2.** Proportion of patients with successful HFNC initiated after a planned extubation, based ROX-HR index at 2 hours (top graph) and 10 hours (bottom graph).

**Table S1.** Comparison of the difference in ROX-HR index over different time points during HFNC.

|  |  | HFNC Success (n = 84) | HFNC failure (n = 61) | p value |
| --- | --- | --- | --- | --- |
| ROX-HR index | Difference between 2 and 6 hours | 0.16 (-1.35 - 1.75) | 0.58 (-0.90 - 1.53) | 0.798 |
|  | Difference between 2 and 10 hours | 1.27 (-1.01 - 3.53) | 0.07 (-1.56 - 1.78) | 0.034 |
|  | Difference between 6 and 10 hours | 0.88 (-0.81 - 2.87) | 0.10 (-1.62 - 0.74) | 0.013 |
|  |  |  |  |  |
| ROX index | Difference between 2 and 6 hours | 0.06 (-0.94 – 1.37) | 0.15 (-0.71 – 1.14) | 0.885 |
|  | Difference between 2 and 10 hours | 1.03 (-0.85 – 2.83) | 0.07 (-0.86 – 1.30) | 0.059 |
|  | Difference between 6 and 10 hours | 0.77 (-0.68 – 2.07) | 0.17 (-0.77 – 0.94) | 0.114 |
|  | | | | |

**Table S2.** Prediction of HFNC failure based on ROX and ROX-HR cut offs at different time points.

| Prediction of HFNC failure for patients initiated on HFNC for acute respiratory failure | | | | | | |
| --- | --- | --- | --- | --- | --- | --- |
|  | Sensitivity (%) | Specificity (%) | PPV (%) | NPV (%) | LR + | LR - |
| 2-hour ROX-HR < 5.00 | 46.50 | 81.48 | 66.67 | 65.67 | 2.51 | 0.66 |
| 2-hour ROX < 5.00 | 53.49 | 75.93 | 63.89 | 67.12 | 2.22 | 0.61 |
| 6-hour ROX-HR < 5.00 | 34.29 | 92.00 | 75.00 | 66.67 | 4.29 | 0.71 |
| 6-hour ROX < 5.00 | 37.14 | 88.00 | 68.42 | 66.67 | 3.10 | 0.71 |
| 10-hour ROX-HR < 5.00 | 34.48 | 93.48 | 76.92 | 69.35 | 5.29 | 0.70 |
| 10-hour ROX < 5.00 | 34.48 | 89.13 | 66.67 | 68.33 | 3.17 | 0.74 |
| 18-hour ROX-HR < 5.00 | 45.00 | 97.50 | 90.00 | 78.00 | 18.00 | 0.56 |
| 18-hour ROX < 5.00 | 30.00 | 92.50 | 66.67 | 72.55 | 4.00 | 0.76 |
| 24-hour ROX-HR < 5.00 | 42.86 | 93.94 | 75.00 | 79.49 | 7.07 | 0.61 |
| 24-hour ROX < 5.00 | 35.71 | 90.91 | 62.50 | 83.33 | 3.93 | 0.71 |
|  |  |  |  |  |  |  |
| Prediction of HFNC failure for patients initiated on HFNC after a planned extubation | | | | | | |
|  | Sensitivity (%) | Specificity (%) | PPV (%) | NPV (%) | LR + | LR - |
| 2-hour ROX-HR < 6.00 | 20.00 | 92.86 | 60.00 | 68.42 | 2.80 | 0.86 |
| 2-hour ROX < 6.00 | 13.33 | 92.86 | 50.00 | 66.67 | 1.87 | 0.93 |
| 6-hour ROX-HR < 6.00 | 18.18 | 92.59 | 50.00 | 73.53 | 2.45 | 0.88 |
| 6-hour ROX < 6.00 | 36.36 | 88.89 | 57.14 | 77.42 | 3.27 | 0.72 |
| 10-hour ROX-HR < 6.00 | 27.27 | 96.15 | 75.00 | 75.76 | 7.08 | 0.76 |
| 10-hour ROX < 6.00 | 36.36 | 92.31 | 66.67 | 77.42 | 4.73 | 0.69 |
| 18-hour ROX-HR < 6.00 | 20.00 | 95.65 | 66.66 | 73.33 | 4.60 | 0.84 |
| 18-hour ROX < 6.00 | 40.00 | 86.96 | 57.14 | 76.92 | 3.07 | 0.69 |
| 24-hour ROX-HR < 6.00 | 33.33 | 95.24 | 75.00 | 76.92 | 7.00 | 0.70 |
| 24-hour ROX < 6.00 | 55.56 | 90.48 | 71.43 | 82.61 | 5.84 | 0.49 |
|  |  |  |  |  |  |  |

HFNC: high flow nasal cannula therapy; PPV: positive predictive value; NPV: negative predictive value; LR: likelihood ratio

**Table S3**. Cox proportional regression analysis evaluating ROX ≥ 4.88 for the likelihood of HFNC failure in patients with acute respiratory failure

|  |  | Univariate analysis | p value | Multivariate analysis | p value |
| --- | --- | --- | --- | --- | --- |
| ROX ≥ 4.88 | 2 hours | 0.461 (0.252-0.843) | 0.012 | 0.437 (0.234-0.818) | 0.010 |
|  | 6 hours | 0.447 (0.225-0.891) | 0.022 | 0.472 (0.226-0.985) | 0.045 |
|  | 10 hours | 0.441 (0.203-0.955) | 0.038 | 0.485 (0.215-1.095) | 0.082 |
|  | 12 hours | 0.713 (0.311-1.634) | 0.436 | 0.992 (0.393-2.502) | 0.987 |
|  |  |  |  |  |  |
| Variables included in the multivariate analysis: gender, Acute physiologic assessment and chronic health evaluation (APACHE) II, Charlson comorbidity index and number of chest x-ray quadrants involved. | | | | | |

**Table S4.** Prediction of HFNC outcomes in patients with acute respiratory failure based on ROX and ROX-HR cut offs at different time points.

| 1. Prediction of HFNC success | | | | | | |
| --- | --- | --- | --- | --- | --- | --- |
|  | Sensitivity (%) | Specificity (%) | PPV (%) | NPV (%) | LR + | LR - |
| 2-hour ROX-HR > 6.80 | 55.56 | 74.41 | 73.17 | 57.14 | 2.17 | 0.60 |
| 2-hour ROX ≥ 4.88 | 75.90 | 51.20 | 66.10 | 62.90 | 1.56 | 0.47 |
| 6-hour ROX-HR > 6.80 | 70.00 | 57.14 | 70.00 | 57.14 | 1.63 | 0.53 |
| 6-hour ROX ≥ 4.88 | 88.00 | 37.10 | 66.70 | 68.40 | 1.40 | 0.32 |
| 10-hour ROX-HR > 6.80 | 78.26 | 58.62 | 75.00 | 62.96 | 1.89 | 0.37 |
| 10-hour ROX ≥ 4.88 | 89.10 | 34.50 | 68.30 | 66.70 | 1.36 | 0.32 |
| 12-hour ROX-HR > 6.80 | 70.21 | 53.57 | 71.74 | 51.72 | 1.51 | 0.56 |
| 12-hour ROX ≥ 4.88 | 85.11 | 28.57 | 66.66 | 53.55 | 1.19 | 0.52 |
|  |  |  |  |  |  |  |
| 1. Prediction of HFNC failure | | | | | | |
|  | Sensitivity (%) | Specificity (%) | PPV (%) | NPV (%) | LR + | LR - |
| 2-hour ROX-HR < 4.50 | 39.53 | 88.89 | 73.91 | 64.86 | 3.56 | 0.68 |
| 2-hour ROX < 2.85 | 4.70 | 100.00 | 100.00 | 56.80 | ∞ | 0.95 |
| 6-hour ROX-HR < 5.00 | 34.29 | 92.00 | 75.00 | 66.67 | 4.29 | 0.71 |
| 6-hour ROX < 3.47 | 11.40 | 96.00 | 66.70 | 60.80 | 2.85 | 0.92 |
| 12-hour ROX-HR < 5.00 | 39.29 | 93.61 | 78.57 | 72.13 | 6.15 | 0.65 |
| 12-hour ROX < 3.85 | 14.29 | 97.87 | 80.00 | 65.71 | 6.71 | 0.88 |
|  |  |  |  |  |  |  |

HFNC: high flow nasal; PPV: positive predictive value; NPV: negative predictive value; LR: likelihood ratio
